# Peritoneal Breach as an Indication for Exploratory Laparotomy in Penetrating Abdominal Stab Injury: Operative Findings in Haemodynamically Stable Patients

**DOI:** 10.1155/2015/407173

**Published:** 2015-05-12

**Authors:** Jasmina Kevric, Victor Aguirre, Kate Martin, Dinesh Varma, Mark Fitzgerald, Charles Pilgrim

**Affiliations:** Emergency & Trauma Centre, The Alfred Hospital, Commercial Road, Melbourne, VIC 3004, Australia

## Abstract

*Introduction*. Management of haemodynamically stable patients with penetrating abdominal injuries varies from nonoperative to operative management. The aim was to investigate whether peritoneal breach when used as an indication for exploratory laparotomy appropriately identified patients with intra-abdominal visceral injury.* Methods.* We conducted retrospective cohort study of all patients presenting with PAI at a major trauma centre from January 2007 to December 2011. We measured the incidence of peritoneal breach and correlated this with intra-abdominal visceral injury diagnosed at surgery.* Results*. 252 patients were identified with PAI. Of the included patients, 71 were managed nonoperatively and 118 operatively. The operative diagnoses included nonperitoneal-breaching injuries, intraperitoneal penetration without organ damage, or intraperitoneal injury with organ damage. The presenting trauma CT scan was reported as normal in 63%, 34%, and 2% of these groups, respectively. The total negative laparotomy/laparoscopy rate for all patients presented with PAI was 21%, almost half of whom had a normal CT scan.* Conclusion*. We found that peritoneal breach on its own does not necessarily always equate to intra-abdominal visceral injury. Observation with sequential examination for PAI patients with a normal CT scan may be more important than exclusion of peritoneal breach via laparoscopy.

## 1. Introduction

Over the last 10 years there has been an increase in stabbing related penetrating abdominal injuries (PAI) [1–3]. Patients who are haemodynamically unstable after PAI are managed surgically with exploratory laparotomy. However, there is controversy regarding the appropriate management of haemodynamically stable patients. Investigations such as local wound exploration (LWE), focused abdominal sonography in trauma (FAST) scan, computer tomography (CT) scan, and diagnostic laparoscopy have been used to evaluate the need for therapeutic intervention and laparotomy [4]. Mandatory laparotomy in all patients presenting with PAI can, however, result in an unacceptably high rate of nontherapeutic surgery, exposing patients to morbidity of up to 20% associated with laparotomy [5]. In high volume centres in the USA and Europe there is a move towards conservative management of more patients with PAI, but in Australia there remains a liberal approach to surgery, with a resultant definably higher rate of nontherapeutic laparotomy [6].

An Australasian survey [7] found agreement among general and trauma surgeons that peritonism and haemodynamic instability warrant laparotomy while the majority of surgeons felt that LWE by an experienced surgeon was most useful in assessing PAI. A valid indication for laparotomy was thought to be a breach in the fascial layer seen on exploration of the wound.

Furthermore, selective nonoperative management (SNOM) was strongly agreed to be an effective and safe way of managing abdominal stab wounds by 75% of British and American general and trauma surgeons recently surveyed [8]. This view has been reflected by increasing rates of SNOM in the practice of many centres in the USA and Europe [9].

The aim of this study was to investigate whether simple peritoneal breach when used as an indication for exploratory laparoscopy or laparotomy at a major Australian trauma centre from January 2007 to December 2011 appropriately identified visceral intra-abdominal injuries.

## 2. Methods

### 2.1. Setting

The state of Victoria, Australia, has one paediatric and two adult Major Trauma Services (MTS) located within metropolitan Melbourne. Major trauma triage guidelines direct 85% of major trauma patients to a MTS for definitive treatment [10]. Alfred Hospital's (as one of the state's MTS) trauma registry prospectively records prehospital and hospital data on all major trauma patients (defined as Injury Severity Score (ISS) greater than 15, requiring urgent surgery, requiring Intensive Care Unit admission, or in-hospital death).

### 2.2. Patient Selection

Patients presenting with PAI from January 2007 to December 2011 were identified from the Alfred trauma registry and objective data on demographics, presenting vital signs and hospital outcomes, were extracted. Patients with gunshot wounds (GSW) and concomitant blunt trauma were excluded. Following identification, a retrospective chart review was conducted and data collected on patient assessment and management. Focused abdominal sonography in trauma (FAST), where performed, included examination of four areas for free fluid: perihepatic and hepatorenal space, perisplenic space, pelvis, and pericardium. This was performed by the treating emergency physician or registrar.

**Figure 1:**
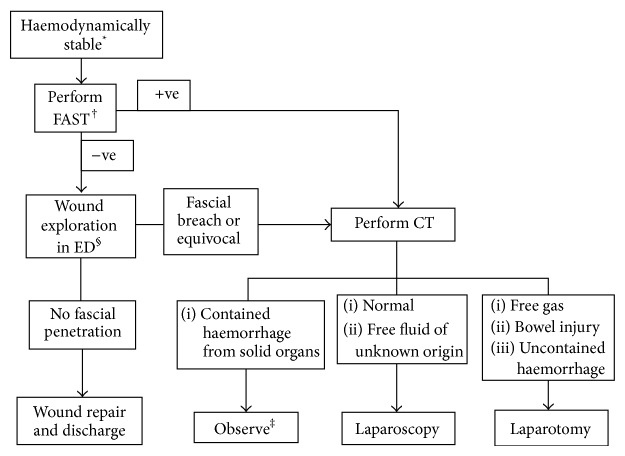
Management of abdominal stab wounds in haemodynamically stable patients. ^*^Haemodynamically unstable patients should have early laparotomy. ^†^FAST can be omitted where not available. ^‡^Observation should be protocolised with regular reviews by experienced personnel ± serial FASTs if available. ^§^Performed by operators with appropriate training.

A positive laparotomy/laparoscopy was defined as an operation involving therapeutic intervention for organ injury. Simple peritoneal breach was not considered a positive operative finding. A negative laparotomy/laparoscopy was defined as nontherapeutic surgical intervention. A patient was deemed to be nonoperatively managed if no surgical intervention or LWE under general anesthesia was undertaken. Haemodynamic stability was defined as a presenting systolic blood pressure of over 90 mmHg. A guideline for the management of patients presenting with PAI is illustrated in Figure 1.

### 2.3. Analysis

Normally distributed continuous variables were presented using mean and standard deviation while ordinal or skewed data were presented using median and interquartile range. Student's *t*-test was used to calculate statistical significance between two means, Wilcoxon Rank Sum test was used for difference between two medians, and the chi-squared test was used for difference between two proportions. The study was approved by The Alfred Hospital Research and Ethics Committee.

## 3. Results

There were 252 patients identified with PAI over the specified study period and only patients with stab wounds were included. Of these, 51 were haemodynamically unstable on arrival and were excluded. Twelve patients presenting after GSW were also excluded, with 189 patients eventually included in the study with characteristics illustrated in Table 1.

Of the 189 included patients, 71 were managed nonoperatively with serial observations and were discharged after 24–48-hour period without any documented adverse events. Within this nonoperatively managed group, 60 (84.5%) patients had a CT scan. The CT scans were reported as normal in 54 (90.0%) of these patients while 6 (10%) CT scans showed small amount of intraperitoneal fluid. This group of patients had a significantly shorter length of stay in hospital. The remaining 118 patients presenting with PAI were managed operatively (Table 2).

Of those patients operatively managed, 12 underwent LWE in theatre of which 10 (66.7%) had a CT scan reported as normal (Table 3). None of these patients were found to have fascial penetration. Twenty patients had diagnostic laparoscopy only, of which 11 (55%) had a normal CT scan. There were 16 patients who underwent laparoscopy converted to laparotomy (five of whom (31.3%) had normal CT imaging) and 70 patients proceeded directly to laparotomy (with further 5 patients (7.1%) having normal CT scans in this subgroup).

The operative diagnoses were classified into 3 groups: superficial or nonperitoneal-breaching injuries (*n* = 27); intraperitoneal penetration without organ damage (*n* = 38); or intraperitoneal injury with organ damage (*n* = 53), Table 2. Patients with superficial or nonperitoneal-breaching injury had undergone a CT scan reported as normal in 94% of cases (*n* = 16). Similarly, in more than one third of patients with peritoneal breach but no intra-abdominal injury, CT scanning had been reported as normal (*n* = 14, 56%). There was only 1 patient who had peritoneal breach and organ injury in whom the CT scan had been reported as normal (1.9%); this patient had a small contained liver haematoma that did not require further intervention.

From the entire cohort of 189 patients, 85 patients had CT scans reported as normal, 54 of whom were treated nonoperatively while 31 had operative management (only 1 of which had intra-abdominal injury).

Table 4 demonstrates the total negative laparotomy/laparoscopy rate for all patients presented with PAI of 33.9% (*n* = 40); almost half of these patients had a normal CT scan (*n* = 16 (40%)). The average length of stay in hospital for the total cohort was 4.1 (1.8–6.8) days with 28 (14.8%) patients admitted to the ICU. Postoperative patients had a hospital stay of 5.7 (3.7–8.8) days compared to 2.3 (0.6–3.5) days for patients that were managed nonoperatively (*P* < 0.05).

## 4. Discussion

The present study highlights the approach of an Australian major trauma hospital to the management of PAI. Mandatory laparotomy for stab wound injuries had been the gold standard since World War I [11]; however there is a shift towards SNOM for haemodynamically stable patients without signs of peritonitis. Peritoneal breach is an indication for laparotomy at our cohort despite other studies suggesting that these patients can be managed conservatively [12]. Procedures such as FAST scan, LWE under GA, CT scan, and diagnostic laparoscopy have been used to make such a diagnosis. However, only 10% of patients with possible peritoneal breach on CT scan were managed conservatively (24–48 hours of observation followed by discharge) indicating a low threshold for operative management. CT scanning remains a critical tool allowing conservative management, and there were 60 CT scans performed amongst the 71 conservatively managed patients (54 scans being completely normal with only 6 showing small amount of free fluid). SNOM may be appropriate management for patients with normal CT scans with studies showing that a delay in laparotomy does not increase the complication rate in the conservatively managed group of patients [13].

Many patients with solid organ injury can be successfully managed conservatively [14]. Complications following SNOM of solid organ injury, such as biloma, urinoma, or abscess, can be managed with percutaneous drainage [15]. Nevertheless, vigilance with frequent clinical examination and a low threshold for repeat imaging are mandatory components of SNOM to diagnose deterioration of patient condition and prevent the devastating consequence of missed injuries.

Rather than proceeding to laparoscopy to exclude peritoneal breach (which may not necessarily be associated with intra-abdominal injury in any case), consideration of conservative management in haemodynamically normal patients with PAI and a normal CT scan may be warranted. Our present study shows that peritoneal breach does not necessarily indicate organ damage as 38 of 91 patients (42%) with peritoneal breach did not have intra-abdominal injury.

Selecting the appropriate group of patients for conservative management leads to overall better patient outcome [4]. Utilising CT scanning to exclude intra-abdominal injury rather than proceeding from laparoscopy to laparotomy (upon diagnosis of peritoneal breach) to exclude injury in a patient with an otherwise normal CT scan may be the more appropriate triage tool to minimise missed injuries and maximise the number of patients successfully conservatively managed.

The negative laparotomy rate of 33.9% may also be due to the practice of mandatory progression to laparotomy when peritoneal breach is suspected on CT scans. Our centre's observed negative laparoscopy/laparotomy rate of 33.9% was comparative to other reports worldwide rate of between 10% and 50% [10]. Nevertheless, given that 40% of patients in the negative laparoscopy/laparotomy group had a normal CT scan there may be the possibility for improving our selection of patients treated conservatively based on this finding and this concept warrants further investigation.

## 5. Study Limitations

This study was limited by its retrospective nature relying on documentation for accurate description of patient management and operative findings. Additionally, the unreliability and lack of consistency of documented physical examination findings prevented us from including these parameters in our analysis. Real time vital sign recordings were not available but could better document clinical progress in these patients. Patients were not followed for long-term complications which would be necessary if steps were undertaken towards wider practice of conservative management. Such analyses could also elucidate the incidence of longer-term complications of surgery in this cohort of patients.

## 6. Conclusion

We found that peritoneal breach on its own does not necessarily mean there is intra-abdominal visceral injury. If all haemodynamically stable patients suffering PAI with a normal CT scan were treated nonoperatively, 20 negative laparoscopies/laparotomies and 10 LWE could have been avoided in this cohort of 118 surgically treated patients (avoiding the morbidity of operation in 25% of those otherwise undergoing surgery). Observation with sequential examination for PAI patients with a normal CT scan may be more important than exclusion of peritoneal breach via laparoscopy.

## Figures and Tables

**Table 1:** Characteristics of the patients included in the study.

Variable	Operative management *N* = 118	Conservative management *N* = 71	*P* value
Age, years Mean ± SD	36.0 ± 16.0	31.5 ± 13.9	0.05

ISS^1^ Median (IQR)	9 (4–14)	2 (1–9.5)	0.01

SBP^2^ on arrival, mmHg Mean ± SD	128 ± 25	128 ± 21	0.09

HR^3^ on arrival, b/min Mean ± SD	96 ± 22	106 ± 19	< 0.01

FAST^4^ scan performed *N* (%)	108 (91.5)	67 (94.4)	0.4

CT scan performed *N* (%)	75 (63.5)	60 (84.5)	< 0.01

LWE^5^ performed (in ED) *N* (%)	8 (6.8)	18 (25.4)	< 0.01

Length of hospital stay, days Median (IQR)	5.7 (3.7–8.8)	2.3 (0.6–3.5)	< 0.01

ICU^6^ admission, hours	*N* = 24	*N* = 4	0.5
Median (IQR)	71.5 (22.5–154)	42.5 (25–71.5)	

^1^Injury Severity Score.

^2^Systolic blood pressure.

^3^Heart rate.

^4^Focussed abdominal sonography for trauma.

^5^Local wound exploration under local anesthesia: on arrival to ED (prior to LWE under GA or operative management).

^6^Intensive Care Unit.

**Table 2:** Characteristics of operative group of patients.

	Nonperitoneal-breaching injury	Peritoneal breach (*N* = 91)	
		Organ injury	No organ injury
	*N* = 27 (22.9%)^1^	*N* = 53 (44.9%)	*N* = 38 (32.2%)

Laparoscopy only + converted	10	9	17

Laparotomy	5	44	21

CT, *N* (%)	17 (63.0)	33 (62.3)	25 (65.8)

FAST scan, *N* (%)	25 (92.6)	48 (90.6)	35 (92.1)

SBP^2^, median ± SD	130 ± 4	122 ± 3	136 ± 5

HR^3^, median ± SD	100 ± 5	93 ± 3	96 ± 3

Hospital LOS^4^ days, median ± SD	2.7 ± 4.3	6.8 ± 7.4	5.1 ± 4.7

^1^Diagnosed either by laparoscopy (*n* = 10), laparotomy (*n* = 5), or LWE (in theatre) (*n* = 12).

^2^Systolic blood pressure.

^3^Heart rate.

^4^Length of stay.

**Table 3:** CT findings in relation to operative findings.

Operative findings	Normal CT	Abnormal CT^1^	*P* value
Nonperitoneal-breaching injury *N* = 17	16 (94%)	1 (6%)	< 0.01

Peritoneum breached with organ injury *N* = 33	1 (3%)	32 (97%)	< 0.01

Peritoneum breached but no organ injury *N* = 25	14 (56%)	11 (44%)	0.01

^1^Abnormal CT: intraperitoneal free fluid and/or organ damage.

**Table 4:** Investigation results and haemodynamics of patients with nontherapeutic operation versus conservative management.

	Positive laparoscopy/laparotomy (*n* = 66)	Negative laparoscopy/laparotomy (*n* = 40)	*P* value	Nonoperative management (*n* = 71)	*P* value
FAST^1^ scan positive *N* (%)	19 (28.8%)	4 (10%)	0.04	5 (7%)	0.01

CT normal (*N*)	1 (1.5%)	16 (40%)	0.01	54	< 0.01

HR^2^ Median (SD)	96 ± 22	95 ± 23	0.8	106 ± 19	< 0.01

SBP^3^ Median (SD)	126 ± 25	132 ± 24	0.2	128 ± 21	0.09

Length of hospital stay Median (IQR)	6 (5–9)	4 (2–8)	0.9	2.3 (0.6–3.5)	< 0.01

^∗^Note: 12 patients underwent wound exploration in OT and were excluded from the above analysis.

^1^Focused abdominal sonography for trauma.

^2^Heart rate.

^3^Systolic blood pressure.
